# Burden of caregivers of patients with neuronopathic and non-neuronopathic Gaucher disease in Japan: A survey-based study

**DOI:** 10.1016/j.ymgmr.2023.100994

**Published:** 2023-08-01

**Authors:** Yuta Koto, Aya Narita, Shinichi Noto, Masafumi Okada, Midori Ono, Terumi Baba, Rieko Sagara, Norio Sakai

**Affiliations:** a Child Healthcare and Genetic Science Laboratory, Division of Health Sciences, Osaka University Graduate School of Medicine, 2-2 Yamadaoka, Suita-shi, Osaka 565-0871, Japan; b Division of Child Neurology, Institute of Neurological Science, Tottori University Faculty of Medicine, 86 Nishi-cho, Yonago-shi, Tottori 683-8503, Japan; c Department of Rehabilitation, Niigata University of Health and Welfare, 1398 Shimami-cho, Kita-ku, Niigata-shi, Niigata 950-3198, Japan; d Real-World Evidence Solutions & HEOR, IQVIA Solutions Japan K.K., 4-10-18 Takanawa, Minato-ku, Tokyo 108-0074, Japan; e Japan Medical Office, Takeda Pharmaceutical Company Limited, 2-1-1 Nihonbashi-Honcho, Chuo-ku, Tokyo 103-8688, Japan

**Keywords:** Caregiver burden, Gaucher disease, Japan, Caregiver Impact Questionnaire, Neuronopathic Gaucher disease, Zarit Caregiver Burden Interview

## Abstract

**Background:**

Gaucher disease (GD), a rare lysosomal storage disorder, is associated with considerable patient and caregiver burden. We examined the applicability of existing caregiver questionnaires and assessed the level of burden in caregivers of patients with GD.

**Methods:**

This cross-sectional, non-interventional study was conducted in Japan. Caregivers of patients with confirmed GD (any type) were recruited (patient association group and referral) for pre-testing (May 2021) or the main survey (October–December 2021). Caregivers completed the Caregiver Impact Questionnaire (CIQ; 30 items) and Zarit Caregiver Burden Interview (ZBI; 22 items) on paper. Total CIQ and ZBI scores and subscores were determined overall and by GD type. Inter-item correlations and test-retest reliability (2 rounds, 2 weeks apart) were calculated. The relationship between caregiving duration and caregiver burden was also analyzed.

**Results:**

Nine caregivers (type 2 [GD2]: *n* = 6; type 3 [GD3]: *n* = 3) and 25 caregivers (type 1 [GD1]: *n* = 2; GD2: *n* = 17; GD3: *n* = 6) completed the pre-test and main survey, respectively. In the main survey, mean total CIQ score, all CIQ subscores (except emotional function), and total ZBI score were highest in caregivers of patients with GD2 compared with caregivers of patients with GD1/GD3. High test-retest reliability (Kappa >0.6) was observed for 15 CIQ items and 16 ZBI items. CIQ and ZBI scores appeared to be positively correlated with each other and negatively correlated with caregiving duration.

**Conclusions:**

The CIQ and ZBI are applicable, reliable measures to assess burden in caregivers of patients with GD in Japan. Caregiver burden was highest in caregivers of patients with GD2 and decreased with caregiving duration.

## Highlights

• Gaucher disease (GD) imposes substantial burden on patients and their caregivers.
• CIQ and ZBI are applicable and reliable to assess caregiver burden for GD in Japan.
• Burden was highest in caregivers of patients with type 2 GD than other GD types.
• Caregiver burden decreased with caregiving duration in type 2 GD.

## 1 Introduction

Rare genetic disorders impose a substantial physical, emotional, social, and financial burden on patients and their families [1,2]. One such rare disorder is Gaucher disease (GD), an autosomal recessive lysosomal storage disorder with multiple clinical phenotypes that exhibit a variety of symptoms, particularly hepatosplenomegaly, hematologic abnormalities, and bone disorders [3,4]. Globally, GD is estimated to occur at a prevalence of 0.9 per 100,000 [5]. GD is generally classified into 3 types based on the presence or absence of neurological symptoms, as well as the typical age of patients and the disease course [3,4]. Type 1 GD (GD1) is non-neuronopathic and is the most common type of GD in most countries [6]. Types 2 and 3 (GD2 and GD3) are neuronopathic GD (nGD) types with symptoms that include abnormal eye movements, seizures, and neurodegeneration; GD2 typically manifests in infancy or early childhood with rapid worsening of symptoms, whereas GD3 is considered a more chronic form of nGD [3,4]. Several studies have reported that patients with GD and their caregivers have reduced health-related quality of life (HRQOL) and experience considerable burden related to the disease and its treatment [[7], [8], [9], [10], [11], [12], [13]]. However, most of the patients in these studies had non-neuronopathic GD1, and little is known about the specific patient and caregiver burdens associated with nGD.

In Japan, the proportion of patients with nGD relative to GD1 is higher than in other countries [5,6,[14], [15], [16]]. More than 50% of Japanese patients with GD have GD2 or GD3 [15] compared with only about 6% in non-Japanese countries [6]. Thus, the need to understand HRQOL and the level of burden in patients with nGD and their caregivers is especially relevant in Japan. We recently developed a patient-reported outcome measure (PROM) that can be tailored for use in patients with any type of GD [17]. The PROM consists of a previous questionnaire developed for GD1 [9] translated into Japanese, together with additional questions specifically about neurological symptoms, which were developed following qualitative interviews with Japanese patients with nGD and their caregivers [18]. In parallel with the PROM development, we surveyed caregivers of patients with GD using 2 standardized measures of caregiver burden: the Caregiver Impact Questionnaire (CIQ) [19], which assesses the caregiver burden of lysosomal storage disorders (metachromatic leukodystrophy and mucopolysaccharidosis), and the Zarit Caregiver Burden Interview (ZBI) [20]. Although these questionnaires are well established, to date there have been limited (ZBI) or no (CIQ) studies that used them in caregivers of patients with GD or in Japan. Therefore, in this study, we aimed to examine the applicability of these questionnaires to these caregivers. The findings presented in this report provide insights into the level of burden in caregivers of patients with GD1, GD2, or GD3.

## 2 Materials and methods

### 2.1 Study design

This was a cross-sectional, non-interventional study conducted in Japan comprising 3 stages. Stage 1 was a qualitative analysis of interviews with patients with nGD conducted in February and March 2021; the results from Stage 1 have been previously reported [18]. Stage 2 was conducted May 10–20, 2021, and included a pre-test of the PROM and caregiver questionnaires and 1:1 interviews to collect participant feedback. Stage 3 was conducted between October 17 and December 31, 2021, and included the main survey of the revised PROM and the caregiver questionnaires. The results of the PROM questionnaire from the pre-test and main survey will be reported separately [17]. The study was conducted in accordance with the protocol, the Declaration of Helsinki, the Guidelines for Good Pharmacoepidemiology Practices, and all applicable laws and regulations. The study was approved by the Osaka University Clinical Research Review Committee (No. 20342). All participants or their legal representative provided written informed consent before study participation. Data collected during the study were de-identified and anonymized.

### 2.2 Study population

Caregivers of patients with GD were recruited in partnership with a patient association group in Japan (Association of Gaucher Disease Patients in Japan), and by referral from leading doctors. Caregivers eligible to participate in this study were persons who, on a daily basis, took care of a child or a family member with a confirmed diagnosis of GD (type 1, 2, or 3) who was receiving treatment. Caregivers of undiagnosed patients, or those who had cognitive disabilities and/or who were not fluent in Japanese, were excluded from the study. Caregivers were included in the study only once, in either the pre-test (Stage 2) or the main survey (Stage 3). Each participant who completed the caregiver questionnaire received a gift card to the value of 2000 Japanese yen.

### 2.3 Questionnaires

Two existing caregiver questionnaires, the CIQ [19] and the validated Japanese version of the ZBI [21], were evaluated and used to assess the burden of GD in caregivers. The CIQ was developed to assess the burden of caregivers of patients with lysosomal storage disease, such as metachromatic leukodystrophy and mucopolysaccharidosis. The questionnaire consists of 30 items in 5 domains, including “social function” (7 items), “impact on daily activities” (5 items), “emotional/psychological function” (10 items), “physical function” (6 items), and “financial impact” (2 items) [19]; the total score ranges from 0 to 120, with a higher score indicating greater burden. The CIQ was translated into Japanese. The ZBI was originally designed to measure the burden of taking care of the elderly [20]; however, it has recently been used in pediatrics to measure the burden of families taking care of children with a disability. The ZBI consists of 22 items, with each item being answered using a 5-point scale (0 [never] to 4 [nearly always]). The total score ranges from 0 to 88, with a higher score indicating greater burden [22]. Item 22 of the ZBI, “Overall, how burdened do you feel in caring for your relative?”, assesses the overall burden of caregiving.

### 2.4 Data collection and interview

Participants completed the caregiver questionnaire once (prior to an online interview) in the pre-test (Stage 2) and twice (2 weeks apart) in the main survey (Stage 3). Paper questionnaires, along with the informed consent form, were mailed to the participants from Osaka University via the patient association group for each round of the main survey. Participants completed the informed consent and the questionnaire, including screening questions (relating to demographics and clinical characteristics). Completed questionnaires and informed consent forms were returned to Osaka University. The study personnel manually entered the data into a pre-configured electronic spreadsheet after checking that the answers were valid.

### 2.5 Statistical analysis

The planned sample size for the pre-test was 20 caregivers. The planned sample size for each round of the main survey was 50, based on a feasibility assessment and the unbalanced distribution of disease types in the Japanese population with GD. All collected data, including total scores and subscores of the CIQ and ZBI, were analyzed by descriptive statistics for all caregivers and for caregivers of each GD type in the pre-test and the main survey. In the main survey, responses from the first round were used for the analysis if caregivers responded to both rounds of the questionnaire. Continuous variables are presented as mean (standard deviation [SD]) and median (minimum, maximum), and categorical variables are presented as frequencies and percentages. Test-retest reliability was evaluated by Kappa coefficients using responses from 2 rounds of the questionnaires in the main survey. Inter-item correlation coefficients were calculated in the pre-test and the main survey for all caregivers and for caregivers of each GD type. Content consistency of the CIQ and ZBI was evaluated by Cronbach's alpha, calculated using all cases with complete answers, for both the pre-test and the main survey. The relationship between caregiving duration and caregiver burden was also analyzed by plotting the caregiving duration against caregiver questionnaire scores for all caregivers and for caregivers of patients with GD2 and GD3; correlation coefficients were also calculated. Similar analyses for caregivers of patients with GD1 were not possible due to the smaller numbers of these caregivers. Uncollected data were not imputed, and no sensitivity analysis was conducted due to the limited sample size. Multiplicity was not applicable for this study. No adjustments for confounding variables or bias were performed. All statistical analyses were conducted using R, Version 4.0.2 (R Foundation for Statistical Computing, Vienna, Austria).

## 3 Results

### 3.1 Caregiver demographics and characteristics

All caregivers who completed the caregiver questionnaires cared for different patients with GD (i.e., none were from the same family) in both the pre-test and the main survey. In the pre-test, a total of 9 caregivers completed the questionnaires (Table 1). Of these, 6 were caregivers of patients with GD2, and 3 were caregivers of patients with GD3. There were no caregivers of patients with GD1. Most caregivers were female, and the mean age was 41.3 years in caregivers of patients with GD2. Data on caregiver age were missing for all 3 caregivers of patients with GD3. Mean duration of caregiving was 5.3 and 16.1 years in caregivers of patients with GD2 and GD3, respectively.

**Table 1:** Caregiver demographics and characteristics.

Characteristics	Pre-test				Main survey^a^, ^b^			
	GD1 (*N* = 0)	GD2 (*N* = 6)	GD3 (*N* = 3)	Overall (*N* = 9)	GD1 (*N* = 2)	GD2 (*N* = 17)	GD3 (*N* = 6)	Overall (*N* = 25)
Sex, *n* (%)								
Male	–	1 (16.7)	0 (0)	1 (11.1)	0 (0)	2 (11.8)	0 (0)	2 (8.0)
Female	–	5 (83.3)	2 (66.7)	7 (77.8)	2 (100)	15 (88.2)	6 (100)	23 (92.0)
Not collected	–	0 (0)	1 (33.3)	1 (11.1)	0 (0)	0 (0)	0 (0)	0 (0)
Age, years								
Mean (SD)	–	41.3 (5.4)	NA	41.3 (5.4)	72.0 (21.2)	39.9 (6.5)	53.3 (15.3)	45.7 (13.8)
Median (min, max)	–	39.5 (37.0, 51.0)	NA	39.5 (37.0, 51.0)	72.0 (57.0, 87.0)	39.0 (31.0, 56.0)	51.5 (38.0, 76.0)	41.0 (31.0, 87.0)
Not collected, *n* (%)	–	0 (0)	3 (100)	3 (33.3)	–	–	–	–
Duration of caregiving, years								
Mean (SD)	–	5.3 (4.4)	16.1 (12.0)	9.3 (9.1)	56.9 (45.1)	9.6 (8.14)	33.3 (19.4)	20.8 (22.2)
Median (min, max)	–	5.2 (0.7, 10.5)	23.0 (2.3, 23.0)	7.0 (0.7, 23.0)	56.9 (25.0, 88.8)	8.8 (0.7, 30.1)	37.5 (9.8, 56.1)	11.0 (0.7, 88.8)
Not collected, *n* (%)	–	1 (16.7)	0 (0)	1 (11.1)	0 (0)	4 (23.5)	0 (0)	4 (16.0)

GD1/2/3, type 1/2/3 Gaucher disease; max, maximum; min, minimum; NA, not applicable; SD, standard deviation.

a Responses from the first round were used if caregivers responded to both rounds of the questionnaire in the main survey.

b Only 1 of the responses was used for 1 caregiver who was a caregiver of 2 patients and responded to the questionnaire twice.

In the main survey, a total of 25 caregivers completed the caregiver questionnaires at least once (Table 1). Of these, 2, 17, and 6 were caregivers of patients with GD1, GD2, and GD3, respectively. Most caregivers were female, and caregivers of patients with GD2 were younger than the caregivers of patients with GD1 or GD3. Mean duration of caregiving was 56.9 years, 9.6 years, and 33.3 years in caregivers of patients with GD1, GD2, and GD3, respectively.

### 3.2 Caregiver questionnaire scores

In the pre-test, mean (SD) total CIQ score was 34.2 (29.5), and the total ZBI score was 24.3 (14.1) for the overall population (Table 2). Mean total CIQ score and the total ZBI score were slightly higher in the caregivers of patients with GD2 than in caregivers of patients with GD3. Similar ZBI_22 (i.e., item 22 of the ZBI, “Overall, how burdened do you feel in caring for your relative?”) scores were observed between caregivers of patients with GD2 and GD3.

**Table 2:** Caregiver Impact Questionnaire (CIQ) and Zarit Caregiver Burden Interview (ZBI) scores.

Domain	Pre-test				Main survey^a^, ^b^			
	GD1 (*N* = 0)	GD2 (*N* = 6)	GD3 (*N* = 3)	Overall (*N* = 9)	GD1 (*N* = 2)	GD2 (*N* = 17)	GD3 (*N* = 6)	Overall (*N* = 25)
CIQ total								
Mean (SD)	–	35.5 (27.9)	31.5 (44.5)	34.2 (29.5)	4.0 (4.2)	37.9 (27.5)	32.5 (19.6)	33.9 (25.9)
Median (min, max)	–	35.5 (5.0, 66.0)	31.5 (0, 63.0)	35.5 (0, 66.0)	4.0 (1.0, 7.0)	37.0 (0, 89.0)	32.5 (6.0, 57.0)	33.0 (0, 89.0)
Not collected, *n* (%)	–	2 (33.3)	1 (33.3)	3 (33.3)	–	–	–	–
CIQ social function								
Mean (SD)	–	6.8 (7.3)	6.0 (8.5)	6.6 (6.9)	1.5 (2.1)	9.9 (7.8)	7.7 (5.7)	8.7 (7.3)
Median (min, max)	–	4.0 (0, 18.0)	6.0 (0, 12.0)	4.0 (0, 18.0)	1.5 (0, 3.00)	9.0 (0, 26.0)	7.0 (0, 17.0)	9.0 (0, 26.0)
Not collected, *n* (%)	–	1 (16.7)	1 (33.3)	2 (22.2)	–	–	–	–
CIQ daily activity								
Mean (SD)	–	4.8 (3.4)	5.0 (7.1)	4.9 (4.0)	0 (0)	6.1 (5.2)	5.8 (6.5)	5.5 (5.5)
Median (min, max)	–	6.0 (0, 9.0)	5.0 (0, 10.0)	6.0 (0, 10.0)	0 (0, 0)	6.0 (0, 16.0)	3.5 (0, 17.0)	4.0 (0, 17.0)
Not collected, *n* (%)	–	1 (16.7)	1 (33.3)	2 (22.2)	–	–	–	–
CIQ emotional function								
Mean (SD)	–	12.4 (11.9)	13.0 (18.4)	12.6 (12.3)	2.5 (2.1)	13.8 (10.5)	13.8 (6.6)	12.9 (9.6)
Median (min, max)	–	7.0 (1.0, 28.0)	13.0 (0, 26.0)	7.0 (0, 28.0)	2.5 (1.0, 4.0)	14.0 (0, 33.0)	13.5 (6.0, 24.0)	13.0 (0, 33.0)
Not collected, *n* (%)	–	1 (16.7)	1 (33.3)	2 (22.2)	–	–	–	–
CIQ physical function								
Mean (SD)	–	5.2 (3.5)	7.0 (6.3)	5.9 (4.4)	0 (0)	6.9 (4.7)	4.5 (3.7)	5.8 (4.7)
Median (min, max)	–	5.0 (1.0, 10.0)	9.0 (0, 12.0)	6.0 (0, 12.0)	0 (0, 0)	7.0 (0, 16.0)	5.5 (0, 8.0)	6.0 (0, 16.0)
Not collected, *n* (%)	–	1 (16.7)	0 (0)	1 (11.1)	–	–	–	–
CIQ financial impact								
Mean (SD)	–	2.3 (2.3)	1.0 (1.7)	1.9 (2.1)	0 (0)	1.4 (2.2)	0.7 (0.8)	1.1 (1.9)
Median (min, max)	–	2.5 (0, 5.0)	0 (0, 3.0)	1.0 (0, 5.0)	0 (0, 0)	0 (0, 7.0)	0.5 (0, 2.0)	0 (0, 7.0)
Not collected, *n* (%)	–	–	–	–	–	–	–	–
ZBI total								
Mean (SD)	–	25.8 (13.9)	21.7 (17.2)	24.3 (14.1)	5.0 (1.41)	23.4 (16.9)	17.8 (14.7)	20.6 (16.3)
Median (min, max)	–	24.0 (6.0, 44.0)	29.0 (2.0, 34.0)	26.5 (2.0, 44.0)	5.0 (4.0, 6.0)	22.0 (0, 56.0)	18.0 (0, 33.0)	21.0 (0, 56.0)
Not collected, *n* (%)	–	1 (16.7)	0 (0)	1 (11.1)	–	–	–	–
ZBI_22^c^								
Mean (SD)	–	1.3 (1.4)	1.3 (1.2)	1.3 (1.2)	0 (0)	1.1 (1.4)	1.2 (0.8)	1.0 (1.2)
Median (min, max)	–	1.0 (0, 3.0)	2.0 (0, 2.0)	1.0 (0, 3.0)	0 (0, 0)	1.0 (0, 4.0)	1.0 (0, 2.0)	1.0 (0, 4.0)
Not collected, *n* (%)	–	–	–	–	–	–	–	–

CIQ, Caregiver Impact Questionnaire; GD1/2/3, type 1/2/3 Gaucher disease; max, maximum; min, minimum; SD, standard deviation; ZBI, Zarit Caregiver Burden Interview.

a Responses from the first round were used if caregivers responded to both rounds of the questionnaire in the main survey.

b Only 1 of the responses was used for 1 caregiver who was a caregiver of 2 patients and responded to the questionnaire twice.

c ZBI_22 is item 22 of the ZBI, “Overall, how burdened do you feel in caring for your relative?”

In the main survey, data from all 25 caregivers who completed the questionnaires at least once were fully collected. Mean (SD) total CIQ score was 33.9 (25.9), and the total ZBI score was 20.6 (16.3) in the overall population (Table 2). Mean total CIQ score and all CIQ subscores (except for the emotional function domain) were highest in caregivers of patients with GD2 compared with caregivers of patients with GD1 or GD3. Moreover, mean total ZBI score was also highest in the caregivers of patients with GD2. However, mean ZBI_22 scores were similar between the disease types. Overall, 12 caregivers (GD1: 2 caregivers; GD2: 7 caregivers; GD3: 3 caregivers) had a ZBI score < 21 (little or no burden), 13 caregivers (GD1: 0 caregivers; GD2: 10 caregivers; GD3: 3 caregivers) had a ZBI score 21–60 (moderate burden), and none had a ZBI score > 60 (severe burden) [20,23].

### 3.3 Test-retest reliability

In the main survey, test-retest reliability was evaluated by calculating the Kappa coefficients from 2 rounds of the caregivers' questionnaire responses. The Kappa coefficients were highest for item 25 of the CIQ (“How often have you been hit, kicked, or bitten by a patient while caring for a patient in the past 7 days?”) (0.916), item 2 of the ZBI (“Do you feel that because of the time you spend with your relative that you don't have enough time for yourself?”) (0.898), and item 24 of the CIQ (“How often in the past 7 days has your sleep been disturbed by the need to care for a patient?”) (0.889) (Table 3). The Kappa coefficients were lowest for item 5 (“How difficult was it for you to spend the past 7 days with family or friends because you had to care for the patient?”) (0.097), item 10 (“How difficult was it for you to go about your daily life at home or work in the past 7 days because you had to care for the patient?”) (0.181), and item 8 (“How often in the past 7 days have you had to interrupt your daily work at home or work because you needed to care for a patient?”) (0.233) of the CIQ. Items 23–30 in the CIQ (i.e., the physical function domain and financial impact domain) generally had better reliability than CIQ items from the other domains. Most items in the ZBI had good reliability, although items 18–21 tended to have a lower reliability than other items.

**Table 3:** Test-retest reliability in the overall caregiver population (Kappa coefficient).

Item	CIQ		ZBI	
	*n*	Estimate (95% CI)	*n*	Estimate (95% CI)
1	23	0.369 (−0.29, 0.767)	23	**0.853 (0.853–0.853)**
2	23	**0.711 (0.711–0.711)**	23	**0.898 (0.898–0.898)**
3	23	**0.715 (0.715–0.715)**	23	**0.709 (0.709–0.709)**
4	23	0.586 (0.586–0.586)	23	**0.615 (0.615–0.615)**
5	22	*0.097 (−1, 1)*	23	**0.746 (0.746–0.746)**
6	22	0.516 (−0.138, 1)	23	**0.838 (0.838–0.838)**
7	22	0.534 (0.204–0.865)	23	**0.760 (0.586–0.933)**
8	22	0.233 (−0.677, 1)	22	0.337 (−0.514, 1)
9	22	0.365 (−0.406, 1)	23	**0.859 (0.859–0.859)**
10	21	0.181 (−1, 1)	23	**0.624 (0.624–0.624)**
11	21	0.295 (−1, 1)	23	**0.713 (0.713–0.713)**
12	22	0.405 (−0.560, 1)	23	**0.710 (0.710–0.710)**
13	22	**0.707 (0.707–0.707)**	23	**0.620 (0.620–0.620)**
14	22	0.578 (0.578–0.578)	23	**0.664 (0.532–0.795)**
15	22	**0.668 (0.668–0.668)**	23	**0.638 (0.638–0.638)**
16	22	**0.753 (0.753–0.753)**	23	0.421 (−1, 1)
17	22	**0.737 (0.737–0.737)**	23	**0.827 (0.827–0.827)**
18	23	**0.692 (0.396–0.989)**	23	0.529 (−0.334, 1)
19	23	0.395 (−0.412, 1)	22	0.557 (0.293–0.821)
20	23	**0.840 (0.840–0.840)**	23	0.557 (0.425–0.689)
21	22	0.540 (0.208–0.871)	23	0.349 (−1, 1)
22	23	0.584 (0.290–0.877)	23	**0.802 (0.802–0.802)**
23	23	**0.801 (0.801–0.801)**	–	**–**
24	23	**0.889 (0.889–0.889)**	–	**–**
25	23	**0.916 (0.916–0.916)**	–	**–**
26	23	**0.772 (0.772–0.772)**	–	**–**
27	23	**0.698 (0.573–0.822)**	–	**–**
28	23	0.593 (0.593–0.593)	–	**–**
29	23	**0.776 (0.776–0.776)**	–	**–**
30	23	**0.687 (0.687–0.687)**	–	**–**

Kappa coefficients > 0.6 are in bold; coefficients < 0.2 are in italics. In general, a Kappa coefficient ≤ 0.2 indicates no to slight agreement, > 0.2 to ≤ 0.4 indicates minimal agreement, > 0.4 to ≤ 0.6 indicates moderate agreement, > 0.6 to ≤ 0.8 indicates substantial agreement, > 0.8 to ≤ 1 indicates almost perfect agreement [31].

CI, confidence interval; CIQ, Caregiver Impact Questionnaire; ZBI, Zarit Caregiver Burden Interview.

### 3.4 Inter-item correlations

Inter-item correlations between CIQ scores, ZBI scores, caregiver's age, and duration of caregiving in the pre-test and the main survey are shown in correlation matrices (Supplementary Fig. 1; Supplementary Fig. 2). In the main survey, positive correlations were observed between total CIQ score, each CIQ subscore, total ZBI score, and ZBI_22 score. These correlations were strong, except for the financial impact domain in the CIQ, which had a weaker correlation with the other items. Furthermore, caregiver age and caregiving duration were negatively correlated with the CIQ and ZBI scores. Inter-item correlations varied between each disease type (data not shown); however, caregiver numbers were too small to draw any conclusions.

### 3.5 Cronbach's alpha

Cronbach's alpha was calculated for the CIQ and ZBI to assess content consistency. Cronbach's alpha was 0.97 for the CIQ (25 caregivers with complete answers) and 0.935 for the ZBI (25 caregivers with complete answers).

### 3.6 Relationship between caregiving duration and caregiver burden

**Fig. 1:**
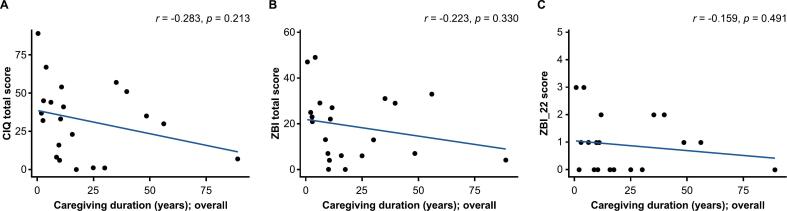
Relationship between caregiving duration and caregiver burden in the overall main survey analysis population. (A) Total CIQ score. (B) ZBI score. (C) ZBI_22 score. The overall main survey analysis population included caregivers of patients with GD1, GD2, and GD3. Caregiving duration, which was assumed to be the same as disease duration, was plotted against the caregiver questionnaire scores. Of the 17 caregivers of patients with GD2 who responded to the questionnaire, caregiver questionnaire scores of 4 caregivers were not plotted because the duration of the caregiving was not accurately collected. ZBI_22 is item 22 of the ZBI, “Overall, how burdened do you feel in caring for your relative?”. CIQ, Caregiver Impact Questionnaire; GD1/2/3, type 1/2/3 Gaucher disease; ZBI, Zarit Caregiver Burden Interview.

**Fig. 2:**
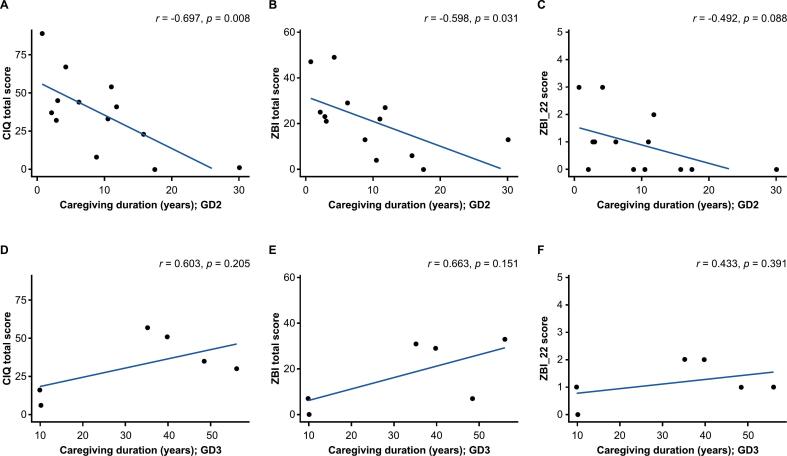
Relationship between caregiving duration and caregiver burden. (A–C) Caregivers of patients with GD2. (D—F) Caregivers of patients with GD3. Caregiving duration, which was assumed to be the same as disease duration, was plotted against the caregiver questionnaire scores in caregivers of patients with GD2 and GD3; no data were plotted for caregivers of patients with GD1 due to limited sample size. Of the 17 caregivers of patients with GD2 who responded to the questionnaire, caregiver questionnaire scores of 4 caregivers were not plotted because the duration of caregiving was not accurately collected. ZBI_22 is item 22 of the ZBI, “Overall, how burdened do you feel in caring for your relative?”. CIQ, Caregiver Impact Questionnaire; GD1/2/3, type 1/2/3 Gaucher disease; ZBI, Zarit Caregiver Burden Interview.

**Fig. 3:**
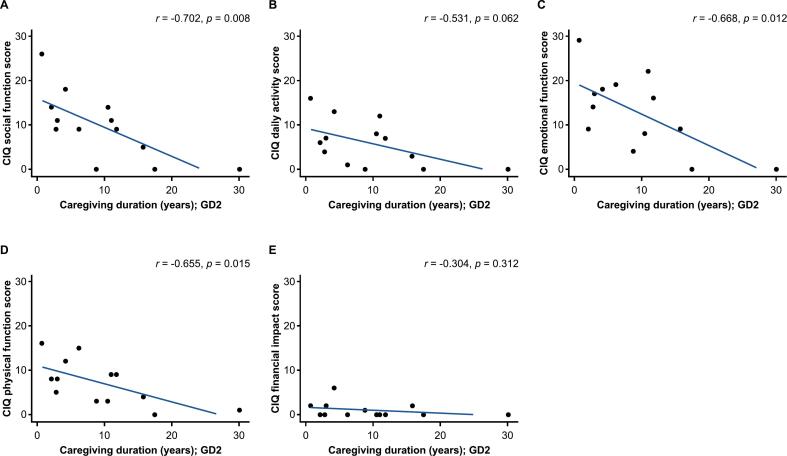
Relationship between caregiving duration and CIQ domains in patients with GD2. (A) CIQ social function. (B) CIQ daily activity. (C) CIQ emotional function. (D) CIQ physical function. (E) CIQ financial impact. Caregiving duration, which was assumed to be the same as disease duration, was plotted against the caregiver questionnaire scores in caregivers of patients with GD2; no data were plotted for caregivers of patients with GD1 and GD3 due to limited sample size.CIQ, Caregiver Impact Questionnaire; GD2, type 2 Gaucher disease.

The relationship between caregiver burden and caregiving duration was evaluated by plotting the caregiver questionnaire scores against duration of caregiving. Overall, although not statistically significant, caregiving duration appeared to show a negative correlation with total CIQ score (*r* = −0.283*; p* = 0.213*)*, total ZBI score (*r* = −0.223; *p* = 0.330), and ZBI_22 score (*r =* −0.159; *p* = 0.491) (Fig. 1). In caregivers of patients with GD2, negative correlations were observed between caregiving duration and total CIQ score (*r* = −0.697; *p* = 0.008), total ZBI score (*r* = −0.598; *p* = 0.031), and ZBI_22 score (*r* = −0.492; *p* = 0.088) (Fig. 2A–C). A similar trend was observed with the CIQ domains, with a strong correlation observed for CIQ social function score, CIQ emotional function score, and CIQ physical function score (Fig. 3). Conversely, in caregivers of patients with GD3, caregiving duration appeared to show positive correlations with total CIQ score (*r* = 0.603; *p* = 0.205), total ZBI score (*r* = 0.663; *p* = 0.151), and ZBI_22 score (*r* = 0.433; *p* = 0.391) (Fig. 2D–F).

## 4 Discussion

This was the first study to evaluate the level of burden in caregivers of patients with GD1, GD2, and GD3 in Japan using the CIQ and ZBI. In the main survey, the overall caregiver burden, assessed by the CIQ and ZBI, was highest in caregivers of patients with GD2 compared with caregivers of patients with GD1 and GD3. Total CIQ score, total ZBI score, and ZBI_22 score appeared to show a negative correlation with caregiving duration, indicating that the burden may decrease with increased duration of caregiving in caregivers of patients with GD2 but not in caregivers of patients with GD3. Furthermore, both caregiver questionnaires had high internal consistency and test-retest reliability, confirming that the CIQ and ZBI are applicable and reliable measures to assess disease burden in caregivers of patients with any type of GD in Japan. Therefore, this study indicates that the CIQ and ZBI may be useful for monitoring caregiver burden over time and for assessing the effects of interventions, such as GD treatments and support systems, in reducing that burden.

Caregiver burden can have a negative impact on the caregiver's physical and mental health, reducing their quality of life [24]. In the main survey, caregiver burden was highest in caregivers of patients with GD2 compared with caregivers of patients with GD1 and GD3. These results were not surprising given that GD2 usually develops in infancy or early childhood and is associated with rapid worsening of severe neuronopathic symptoms [3,4]. The inter-item correlation analysis demonstrated a negative correlation between caregiver age and the level of caregiver burden. Results indicated that the younger age of caregivers of patients with GD2 compared with caregivers of patients with the other GD types potentially contributed to this observation, suggesting that caregiver age is closely associated with caregiver burden. Moreover, similar trends were observed from the results of the PROM scores, with the highest overall disease burden being observed in patients with GD2 [17]. Therefore, the results from this study suggest that caregiver burden is also associated with disease severity and patient burden. Collectively, although statistical tests were not performed due to the small sample size, these results provide important insights into the burden of caregivers of patients with different types of GD. Further studies confirming the difference in caregiver burden scores between caregivers of patients with different GD types and the factors that may be associated with caregiver burden may be required.

In this study, both caregiver questionnaires showed high internal consistency and test-retest reliability. In particular, the test-retest reliability revealed that CIQ items in the physical function and financial impact domains generally have better reliability than items in the other domains. This may be because these 2 domains are generally more stable domains; therefore, caregivers' responses were less likely to change drastically or vary substantially in the 2 weeks between the 2 rounds of surveys. Removal of question items with low reliability may be considered after assessing the background factors that may have influenced the reliability. The total ZBI score ranges from 0 to 88, with a score < 21 generally considered to indicate little or no burden, and a score > 61 indicating severe burden [20,23]; however, cutoff values of the ZBI score for GD have not yet been determined. In this study, the mean (SD) total ZBI score was 20.6 (16.3) in the overall caregiver population, 23.4 (16.9) for caregivers of patients with GD2, and 17.8 (14.7) for caregivers of patients with GD3; approximately half of caregivers had a total ZBI score < 21, and half had a score 21–60, but no caregivers had a total ZBI score > 61 (Fig. 2 and Fig. 3). These scores were lower than reported previously for GD and other rare diseases in other countries; mean ZBI score was 48.6 for caregivers of patients with GD (all types) in China [10], 53.8 and 43.9 for caregivers of patients with mitochondrial disease and intractable epilepsy, respectively, in Korea [25], and 29.0 in caregivers of patients with Duchenne muscular dystrophy in Germany, Italy, the UK, and the USA [26]. However, the scores in the present study were similar to the ZBI scores in the Japanese version reported previously in caregivers of children with tracheostomy (34.7) [27] and in caregivers of children with conditions associated with physical disabilities such as cerebral palsy and epilepsy (28.0) [28]. These results may reflect the characteristics of the Japanese people, who tend to endure and persevere through difficult situations, and who may have rated their level of burden lower than non-Japanese caregivers, despite having a high burden to care for their patients. Therefore, the results from this study confirmed that burden clearly exists and is not low in caregivers of patients with nGD. Furthermore, different factors—such as patient age, extent of disease progression, and the availability and extent of treatment—and government and support systems presumably have affected the level of burden. Therefore, factors that may affect the burden of caregivers should be examined when planning support measures and policies in the future.

Consistent with a study that reported a negative correlation between disease burden and disease duration in patients with GD2 [17], in this study, caregiver burden showed a negative correlation trend with caregiving duration, which was assumed to be the same as disease duration, in caregivers of patients with GD2. These results may be explained by the fact that GD2 is a more severe form of GD that typically affects infants [3,4]. A previous nationwide survey in Japan revealed that patients with GD2 required advanced medical interventions, with about 20% receiving nasal or enteral nutrition and 56.3% having tracheostomy; mechanical ventilation was also used [15]. High caregiver burden would therefore be expected initially with the addition of a new responsibility that the caregivers are unfamiliar with. This may require the caregivers to change their lifestyle to take care of their patient; however, as duration of the disease and duration of caregiving increase, caregivers become more familiar with the disease and its treatment and adapt to giving care. In contrast to the results observed in caregivers of patients with GD2, a positive correlation trend between caregiver burden scores and caregiving duration was observed in caregivers of patients with GD3. Although clinically heterogenous, GD3 is a subacute or chronic form of nGD, with symptoms progressing more slowly than GD2 [3,4]. Therefore, increased caregiver burden with increased caregiving duration may be due to the slower appearance of symptoms and the need for treatment as the disease progresses. Additionally, because patients with GD3 have the prospect of relatively prolonged survival, caregivers may be concerned about ensuring the provision of continued care if they themselves become ill or disabled, which may also explain the results observed. Nonetheless, these results indicated the importance of early recognition of caregiver burden; the needs of the caregivers should be assessed, and social support should be provided early after disease diagnosis to minimize the burden and maintain caregivers' quality of life as much as possible. Further qualitative research, similar to that undertaken for another lysosomal storage disease, acid sphingomyelinase deficiency [29], would provide additional insights into the needs of caregivers and could lead to the development of a GD-specific caregiver burden questionnaire.

This study has some limitations. Unlike international collaborative studies, such as those that might arise from the Gaucher Registry for Development, Innovation & Analysis of Neuronopathic disease initiative, a global nGD patient-driven disease registry that includes data of patients (and their caregivers) with GD [30], the sample size of caregivers included in this study was small; in particular, there were only 2 caregivers of patients with GD1 who completed the questionnaire in the main survey. This is because GD is a rare disease, with an estimated total of 211 patients in Japan [15]; however, caregivers were recruited via a patient association group and by referral from leading doctors, which enabled us to maximize the sample size for this study as much as possible. This study only included caregivers of patients with GD in Japan; therefore, the generalizability of the study results for non-Japanese caregivers may also be limited. Moreover, the duration of disease was considered as being equivalent to the duration of caregiving; we assumed that these would likely be the same in most cases, although it is theoretically possible for these durations to be different. Finally, detailed information on caregivers' demographics and characteristics (e.g., extent of caregiving), as well as patient characteristics (e.g., treatment), was not described or will be reported separately [17].

In conclusion, this study showed that caregiver burden was highest in caregivers of patients with GD2 compared with caregivers of patients with GD1 and GD3. Moreover, caregiver burden decreased with increased duration of caregiving of patients with GD2; therefore, burden should be assessed in caregivers of patients with GD to provide support measures early and in an effective manner, which may help reduce burden and improve caregiver quality of life.

## Funding

This study was funded by Takeda Pharmaceutical Company Limited. Under the direction of the authors, medical writing assistance was provided by Rebecca Lew, PhD, CMPP, and Hana Nomura, BPharm (Hons), of ProScribe – Envision Pharma Group, and was funded by Takeda Pharmaceutical Company Limited. ProScribe's services complied with international guidelines for Good Publication Practice.

## Role of the sponsor

Takeda Pharmaceutical Company Limited was involved in the study design, data collection, data analysis, and preparation of the manuscript. IQVIA Solutions Japan K.K. was responsible for all operational aspects of this study, including study set-up (production of study protocol, informed consent, data collection forms, etc.), data collection set-up (survey distribution, etc.), data analysis, and study reporting.

## Role of contributors

YK was involved in the study design, data collection and analysis, and data interpretation. AN was involved in the study design and data interpretation. SN was involved in the study design and data interpretation. M. Okada was involved in the data analysis. M. Ono was involved in the study design and data interpretation. TB was involved in the study design and data interpretation. RS was involved in the study design and data interpretation. NS was involved in the study design and data interpretation. All authors participated in the drafting, critical revision, and approval of the final version of the manuscript.

**Other contributors/acknowledgments**.

We would like to extend our heartfelt gratitude to all the patients and their caregivers who participated in this study, to the Association of Gaucher Disease Patients in Japan, to Yayoi Hoshi (IQVIA Solutions Inc.), who oversaw the study, and to Sayaka Takabuchi (Osaka University Graduate School of Medicine, Child Healthcare and Genetic Science Laboratory, Division of Health Sciences), who contributed to the study management.

## Declaration of Competing Interest

YK, AN, and NS have received research funding from and have served as consultants and on speaker's bureaus for Takeda Pharmaceutical Company Limited (hereafter Takeda). SN has served as a consultant for Takeda. M. Okada is a former employee of IQVIA Solutions Japan K.K. M. Ono is an employee, and TB and RS are former employees, of Takeda. M. Ono is a stockholder of Takeda.

## Data Availability

The data generated and/or analyzed during the current study are available from the corresponding author on reasonable request.

## Appendix A Supplementary data

Supplementary material Image 1
