# Supplementary material for: Burden of caregivers of patients with neuronopathic and non-neuronopathic Gaucher disease in Japan: A survey-based study
Source: Mol Genet Metab Rep. 2023 Aug 1;36:100994. doi: 10.1016/j.ymgmr.2023.100994 (PMC10412861; doi:10.1016/j.ymgmr.2023.100994)
Supplement: Supplementary file 1 — Supplementary material [file mmc1.docx]

SUPPLEMENTARY MATERIAL

Burden of caregivers of patients with neuronopathic and non-neuronopathic Gaucher disease in Japan: A survey-based Study

Yuta KOTO^a,1^, Aya NARITA^b^, Shinichi NOTO^c,^*, Masafumi OKADA^d^, Midori ONO^e^, Terumi BABA^e^, Rieko SAGARA^e^, Norio SAKAI^a^

**AUTHORS’ AFFILIATIONS**

^a^ Child Healthcare and Genetic Science Laboratory, Division of Health Sciences, Osaka University Graduate School of Medicine, 2-2 Yamadaoka, Suita-shi, Osaka, 565-0871, Japan

^b^ Division of Child Neurology, Institute of Neurological Science, Tottori University Faculty of Medicine, 86 Nishi-cho, Yonago-shi, Tottori, 683-8503, Japan

^c^ Department of Rehabilitation, Niigata University of Health and Welfare, 1398 Shimami-cho, Kita-ku, Niigata-shi, Niigata, 950-3198, Japan

^d^ Real-World Evidence Solutions & HEOR, IQVIA Solutions Japan K.K., 4-10-18 Takanawa, Minato-ku, Tokyo, 108-0074, Japan

^e^ Japan Medical Office, Takeda Pharmaceutical Company Limited, 2-1-1 Nihonbashi-Honcho, Chuo-ku, Tokyo, 103-8688, Japan

Present address

^1^ Graduate School of Nursing, Faculty of Nursing, Kansai Medical University, 2-2-2, Shinmachi, Hirakata-shi, Osaka, 573-1004, Japan

*** AUTHOR FOR CORRESPONDENCE**

Shinichi Noto

Department of Rehabilitation

Niigata University of Health and Welfare

1398 Shimami-cho, Kita-ku, Niigata-shi, Niigata, 950-3198, Japan

Tel: +81-2-5257-4733

Email: noto@nuhw.ac.jp

**Supplementary Fig. 1.** Inter-item correlations of caregiver questionnaires in the overall main survey analysis population. The overall main survey analysis population included caregivers of patients with GD1, GD2, and GD3. The magnitude of the correlation coefficients is indicated by the color. A positive correlation is indicated in blue and a negative correlation is indicated in red. ZBI_22 is item 22 of the ZBI, “Overall, how burdened do you feel in caring for your relative?”. CIQ, Caregiver Impact Questionnaire; GD1/2/3, type 1/2/3 Gaucher disease; ZBI, Zarit Caregiver Burden Interview.


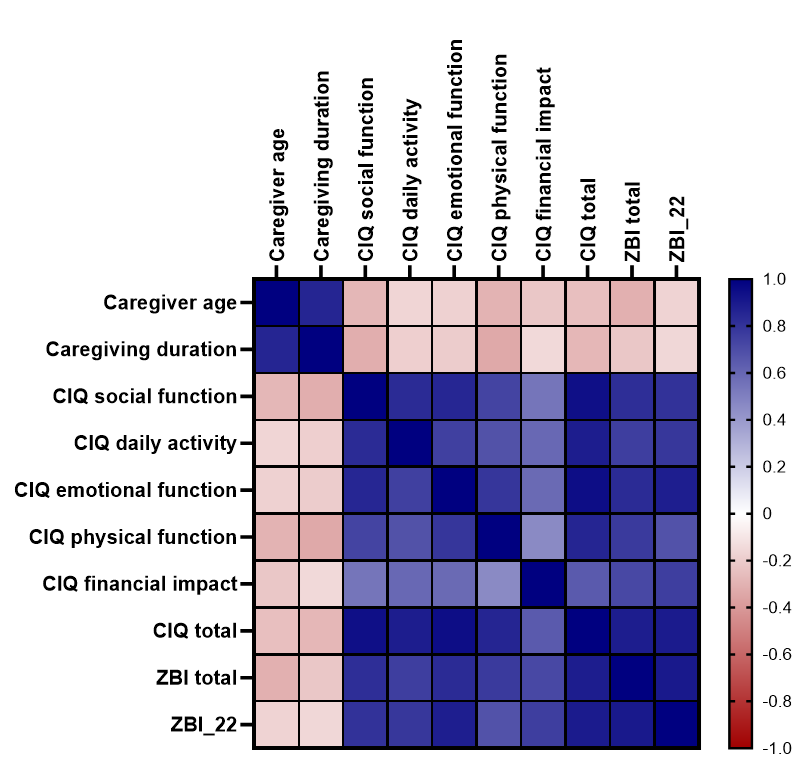


**Supplementary Fig. 2.** Inter-item correlations of caregiver questionnaires in the overall pre-test analysis population. The overall pre-test analysis population included data of caregivers of patients with GD2 and GD3. The magnitude of the correlation coefficients is indicated by the color. A positive correlation is indicated in blue and a negative correlation is indicated in red. ZBI_22 is item 22 of the ZBI, “Overall, how burdened do you feel in caring for your relative?”. CIQ, Caregiver Impact Questionnaire; GD2/3, type 2/3 Gaucher disease; ZBI, Zarit Caregiver Burden Interview.
